# Accuracy of iohexol plasma clearance for GFR-determination: a comparison between single and dual sampling

**DOI:** 10.1186/s12882-018-0965-7

**Published:** 2018-07-11

**Authors:** Yong Zhang, Zhun Sui, Ze Yu, Tai Feng Li, Wan Yu Feng, Li Zuo

**Affiliations:** 1 Department of Nephrology, Peking University People’s Hospital, No. 11 Xizhimen South Street, Xi Cheng District, Beijing, 100044 China; 2 Department of Pharmacy, Peking University People’s Hospital, No. 11 Xizhimen South Street, Xi Cheng District, Beijing, China

**Keywords:** Glomerular filtration rate, Plasma clearance, Iohexol, Single sample, Slope-intercept

## Abstract

**Background:**

Current guidelines regarding plasma-sampling techniques for glomerular filtration rate (GFR) determination are inconsistent. Single-sample methods are commonly believed not to be precise enough to meet clinical demands. The present study compared the agreement between single- and dual- plasma sampling methods with a three-point plasma clearance of iohexol.

**Methods:**

A total of 46 healthy volunteers and 124 chronic kidney disease (CKD) patients with varying degrees of renal dysfunction received 5 ml iohexol (300 mgI/ml) i.v. and plasma samples were drawn at 2-, 3- and 4-h post-injection. Plasma-iodine concentrations were detected by high-performance liquid chromatography (HPLC).

**Results:**

Bias was similar among single-plasma sampling methods (SPSM) and dual-plasma sampling methods (DPSM). The best correlation was obtained from the 2- and 4-h DPSM (concordance correlation coefficient [CCC]: 0.9988) with none of the estimates differed by more than 30% from the reference GFR and only one (0.06%) estimate differed by more than 10% (P_30_, 100%; P_10_, 99.4%). SPSM using samples around 3- or 4-h demonstrated acceptable accuracy at a GFR level of ≥60 ml/min/1.73m^2^ (P_30_ = 100% and P_10_ > 75% for both measurements).

**Conclusion:**

4-h SPSM is advantageous in clinical practice in subjects with GFR ≥ 60 ml/min/1.73m^2^. For patients with an expected GFR < 60 ml/min/1.73m^2^, a prolonged sampling time is more reliable.

## Background

Calculated plasma clearance of iohexol after a single bolus injection correlates well with the “gold standard of renal function” inulin clearance and is recommended as a robust standard for evaluating renal function [1–3]. The mathematical model for the drug elimination curve is an open two-compartment system. The exogenous tracer for glomerular filtration rate (GFR) measurement is injected in the first compartment, equilibrates with the second compartment, and is excreted from the first compartment by glomerular filtration [4]. However, multiple blood samples beyond 6 h post-injection are needed to calculate the area under the time-concentration curve (AUC), if maximal precision and accuracy of the measurements are sought [5, 6], which would be rather time-consuming and therefore compromise recruitment and subject compliance. A growing need for simplification has led to the application of single-compartment models that need only two to three blood samples, which calculate AUC from the intercept and slope of the final slow disappearance curve. By the two-compartment correction introduced by Brochner-Mortensen [7], the slope-intercept method provides greater simplicity and sufficient accuracy to meet clinical demands.

Further simplified techniques requiring only one blood sample have also been developed [8–14]. Single-plasma sampling methods (SPSM) demonstrate acceptable accuracy, given that the single sample is drawn at a proper time point and there is knowledge about the distribution volume of the injected tracer. However, this method lacks the opportunity for quality control and has been reported inferior to dual-plasma sampling methods (DPSM) [9, 15–18].

In the present study, we compared the clearance values based on DPSM and SPSM with a three-point plasma clearance of iohexol (3 pt. iGFR), to specify whether 3 pt. iGFR measurement can be substituted by DPSM or SPSM, or which estimate is more adequate to be recommended in routine practice.

## Methods

A total of 170 participants (89 females, 52.4%) including 46 healthy volunteers and 124 CKD patients with varying degrees of renal dysfunction were recruited in this study. Mean age was 43 years (range 21–87 years). The mean body-mass index and body-surface area were 24.2 ± 4.0 kg/m^2^ and 1.74 ± 0.2 m^2^ respectively. Mean serum-creatinine was 111.3 μmol/l (range 37.1–797.4 μmol/l). A large proportion of the studied patients were diagnosed with diabetes (14.1%) or hypertension (35.9%).

The participants were examined in a non-fasting state. Baseline blood sample was obtained at test day. A single bolus injection of 5 ml iohexol (Omnipaque, 300 mgI/ml, GE healthcare, Shanghai, China) was given at one side of the upper limb peripheral vein and then blood sample was drawn from the contralateral arm at 2 h, 3 h and 4 h after injection. Plasma-iodine concentrations were detected by high-performance liquid chromatography (Waters Alliance HPLC, Milford, USA).

The protocol was approved by the local ethics committee. All volunteers were informed and signed the consent form.

### Dual- and multi-sample methods (*Cl*_*slope*_)

Based on the one-compartment model, plasma clearance of iohexol was calculated using the slope and the intercept of the regression equation in the final slow clearance.

$$ {\mathrm{C}\mathrm{l}}_{\mathrm{slope}}={\mathrm{Q}}_0/{\mathrm{C}}_1/\mathrm{b} $$

where *Q*_*0*_ is the total injected amount of tracer (mg), *C*_*1*_ and *b* are the intercept and the slope of the linear regression equation between plasma concentration and time (*t*), respectively.

The area under the curve (C_1_/b) calculated by this algorithm was underestimated; therefore, the calculated clearance value was corrected by the Brochner-Mortensen’s formula and standardized for a body-surface area (BSA) of 1.73 m^2^ (the Haycock’s formula) to derive the final GFR [7, 19].

### Single-sample method (*Cl*_*ss*_)

The formula described by Jacobsson [8] was based on corrections for non-immediate mixing and non-uniform distribution of the tracer. The distribution volume was calculated as a function of the body weight.

$$ {Cl}_{ss}=\frac{1}{\mathrm{t}/\mathrm{V}+0.0016}\times \ln \left(\frac{{\mathrm{Q}}_0}{\mathrm{V}\times {\mathrm{C}}_{\mathrm{t}}}\right) $$

where *Q*_*0*_ is the total injected amount of tracer (mg), *t* is the time interval between injection and sampling (min), *C*_*t*_ is the iodine concentration in the plasma sample taken at the time (*t*), and *V* is the calculated distribution volume (ml) of the participant. The clearance values were adjusted to 1.73 m^2^ body surface.

The reference values of GFR were measured as the 2-, 3- and 4-h three-point plasma clearance of iohexol standardized to 1.73 m^2^ BSA (3 pt. iGFR), as described above, also denoted as mGFR (measured GFR). DPSM-GFR and SPSM-GFR were expressed as GFR*ix* or GFR*i*, *i* and *x* impliy the sampling time (hr) of the blood used to calculate the corresponding plasma clearance of iohexol. For example, DPSM-GFR determined by the 2- and 3-h samples was denoted as GFR23, and SPSM-GFR determined by the single 2-h sample was denoted as GFR2, and so on.

### Statistical analysis

Baseline characteristics are presented as the mean ± SD for continuous variables and as n (%) for categorical variables.

Taking 3 pt. iGFR as the reference standard (mGFR), bias was assessed as the median difference and precision was assessed as the interquartile range (IQR) for the difference. Accuracy was assessed as the percentage of estimates that differed within 30 and 10% of the mGFR (i.e. P_30_ and P_10_). Agreement between GFR*ix* and mGFR was reported according to Lin’s concordance correlation coefficient (CCC) [20]. Moreover, the agreement between different methods was evaluated graphically by plotting the ratios of GFR*ix*/mGFR against mGFR according to Bland and Altman [21].

Confidence intervals (CI) were calculated by means of bootstrap methods (1000 bootstraps). The significance of the differences among different methods was determined with the use of the signed-rank test for bias, the bootstrap method for the interquartile range from the 1000 bootstrap samples, and McNemar’s test for P_30_ and P_10_. *P* values < 0.05 were considered significant. Analyses were performed with the use of SPSS for Windows 21.0 (SPSS Inc., USA) and MedCalc version 18 (Medcalc software, Belgium).

## Results

The detailed results of each different sampling method with individual correlations against mGFR are shown in Table 1. Overall, the best correlation from the analyses carried out was obtained from GFR24, the 2- and 4-h blood sampling, with a CCC of 0.9988, which indicates almost perfect agreement with the 3 pt. iGFR. Moreover, GFR24 showed the best accuracy: none of the estimates differed by more than 30% from the mGFR and only one (0.06%) estimate differed by more than 10% (P_30_, 100%; P_10_, 99.4%). Bias was very low and similar among DPSM-GFRs and SPSM-GFRs, while the precision of GFR24 was significantly better with an IQR of the difference of 1.52 ml/min/1.73m^2^.

**Table 1:** Summary of results of correlations between different sampling methods and three-point plasma clearance of iohexol

Method	Difference		P_30_ (95% CI)	P_10_ (95% CI)	Correlation coefficient
	Median (95% CI)	IQR (95% CI)			
GFR23	− 0.1 (− 0.48,-0.02)	3.2 (1.95–4.47)	98.2 (95.9100)	83.5 (77.7,88.8)	0.9893
GFR34	0.29 (− 0.12,-0.80)	5.95 (3.28,8.33)	96.5 (93.5,98.8)	78.8 (72.4,84.7)	0.9678
GFR24	−0.31 (− 0.61,-0.06)	1.52 (1.09,1.90)	100 (100,100)	99.4 (98.24,100)	0.9988
GFR2	0.62 (−0.43,1.62)	6.02 (4.04,9.74)	84.1 (78.8,89.4)	68.2 (61.2,75.3)	0.9533
GFR3	0.57 (−-0.43,1.62)	7.25 (4.90,9.92)	90.6 (85.9,94.7)	74.7 (68.2,81.2)	0.9759
GFR4	−0.98 (−1.49,-0.09)	7.11 (5.07,10.65)	94.1 (90.6,97.1)	76.5 (70.0,82.4)	0.9716

The difference is calculated as GFR*ix*-mGFR, *i* and *x* implies the sampling time (hr) of the blood used to calculate the corresponding plasma clearance, mGFR is defined as the three-point (2-, 3- and 4-h) plasma clearance of iohexol standardized to body-surface area (3 pt. iGFR). P_30_ and P_10_ indicates the percentage of estimates that differed within 30 and 10% of the mGFR. Units are in *ml/min/1.73m*^*2*^. Correlation coefficient was reported according to Lin’s concordance correlation coefficient (CCC)

**Fig. 1:**
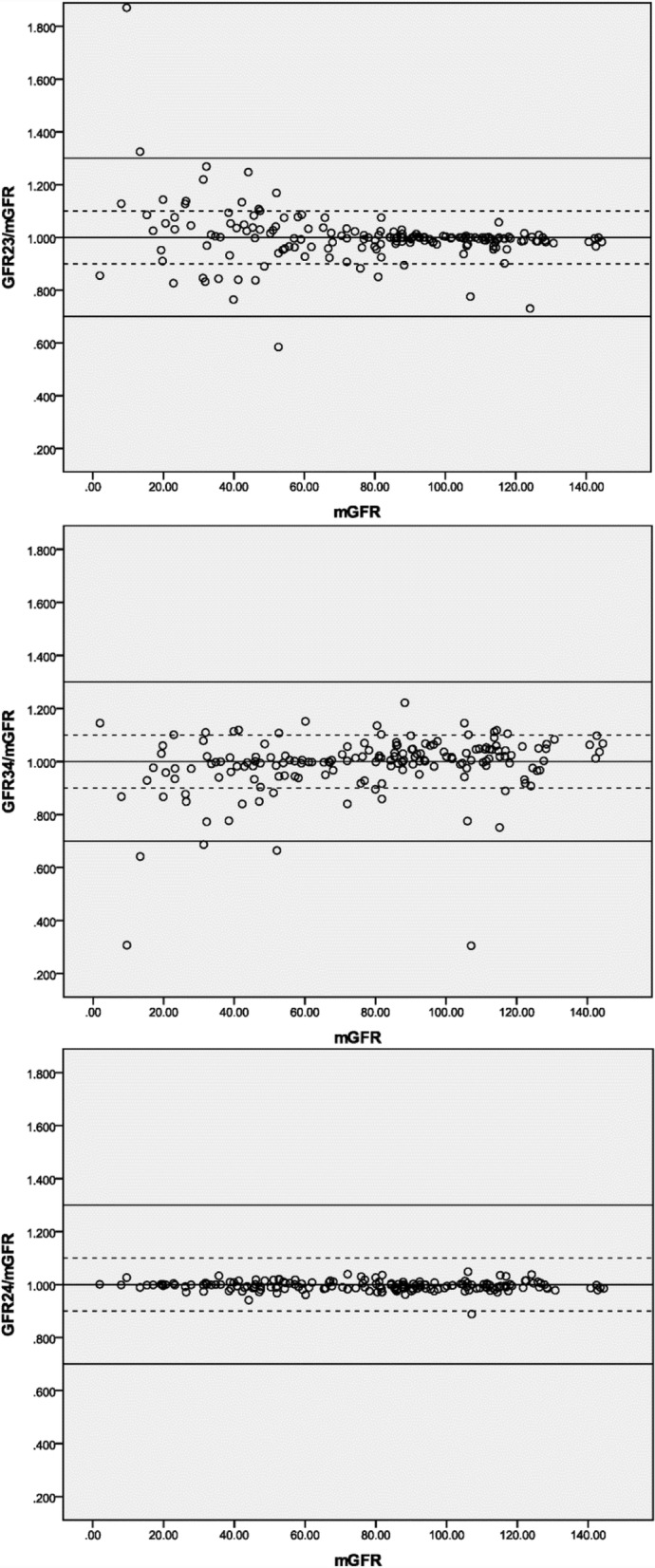
Agreement between DPSM and three-point plasma clearance. *Notes*: The solid lines and the dashed lines delineate the boundaries defined by P_30_ and P_10_, respectively. GFRix: the corresponding DPSM-GFR determined by two samples drawn at the time i and x (hr) after injection. mGFR: the 2-, 3- and 4-hr three-point plasma clearance of iohexol. Units are in *ml/min/1.73m*^*2*^

**Fig. 2:**
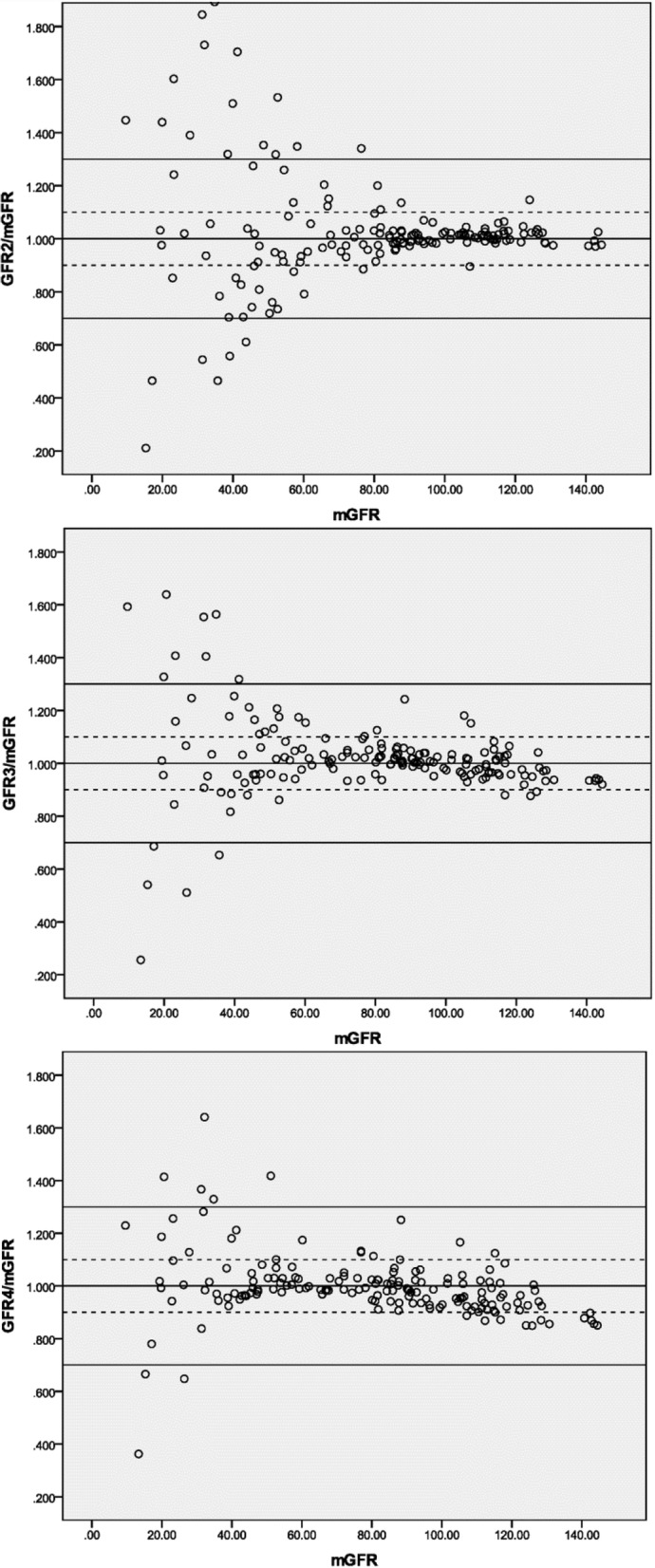
Agreement between SPSM and three-point plasma clearance. *Notes*: The solid lines and the dashed lines delineate the boundaries defined by P_30_ and P_10_, respectively. GFRi: the corresponding SPSM-GFR determined by single sample drawn at the time i (hr) after injection. mGFR: the 2-, 3- and 4-hr three-point plasma clearance of iohexol. Units are in *ml/min/1.73m*^*2*^

The agreement of each sampling method predicting mGFR is graphically illustrated in Figs. 1 and 2, with the solid lines and the dashed lines delineating the boundaries defined by P_30_ and P_10_, respectively. The percentage difference of GFR24 from the mGFR was fairly stable throughout the whole range of measured GFR. With respect to the other methods, the dots become increasingly scattered as GFR decreases, especially the SPSM-GFRs at a GFR level of < 60 ml/min/1.73m^2^.

Table 2 shows the performance of the DPSM-GFRs and SPSM-GFRs at different measured GFR levels. Generally, the correlation metrics were not significantly different between DPSM and SPSM when GFR was above 60 ml/min/1.73m^2^. However, SPSM showed noticeably imprecise and poor accuracy compared to DPSM in the GFR < 60 ml/min/1.73m^2^ subgroup.

**Table 2:** Performance of dual- and single- sampling methods at different GFR levels^a^

Variable	mGFR (*ml/min/1.73m*^*2*^)		
	< 60 (*n* = 58)	60–89 (*n* = 42)	> 90 (*n* = 70)
Bias ─ Mean Difference (95% CI)			
GFR23	1.05 (−0.10,1.7)	−0.17 (− 1.29,0.11)	−0.47 (− 1.10,-0.10)
GFR34	− 0.75 (−1.3,-0.19)	0.35 (− 1.29,1.63)	2.71 (0.83,4.85)
GFR24	− 0.02 (− 0.12,0.02)	−0.73 (− 1.15,0.09)	−0.86 (− 1.30,-0.28)
GFR2	− 0.88 (−4.39,4.51)	0.04 (− 1.64,2.27)	1.19 (− 0.04,1.75)
GFR3	1.31 (− 1.38,4.69)	1.76 (0.91,2.74)	−1.73 (− 3.54,0.26)
GFR4	0.43 (− 0.57,1.74)	0.40 (− 0.95,1.83)	−5.89 (−7.95,3.76)
Precision ─ IQR (95% CI)			
GFR23	5.56 (1.82,8.73)	3.92 (1.25,7.18)	1.79 (1.30,3.03)
GFR34	3.59 (1.36,7.21)	3.75 (1.39,9.56)	7.02 (4.64,12.64)
GFR24	0.71 (0.21,1.39)	1.79 (0.88,3.10)	1.84 (1.17,2.89)
GFR2	21.99 (9.43,32.91)	5.35 (3.24,12.48)	3.45 (2.16,5.02)
GFR3	9.94 (6.26,14.68)	4.68 (1.36,6.84)	6.99 (4.15,11.14)
GFR4	4.93 (2.58,9.27)	3.89 (2.40,12.12)	9.18 (4.96,14.11)
Accuracy^b^ ─ % (95% CI)			
P_30_			
GFR23	96.8 (87.9100)	100^c^	100^c^
GFR34	91.4 (82.8,98.3)	100^c^	98.6 (95.7100)
GFR24	100^c^	100^c^	100^c^
GFR2	55.2 (43.1,69.0)	97.6 (92.9100)	100^c^
GFR3	72.4 (60.3,84.5)	100^c^	100^c^
GFR4	82.8 (72.4,91.4)	100^c^	100^c^
P_10_			
GFR23	60.34 (46.6,72.4)	92.9 (85.7100)	97.1 (92.9100)
GFR34	65.5 (53.5,77.6)	83.3 (71.4,92.9)	87.1 (78.6,94.3)
GFR24	100^c^	100^c^	98.6 (95.7100)
GFR2	25.9 (13.8,36.2)	78.6 (66.7,90.5)	97.1 (92.9100)
GFR3	41.4 (29.3,55.2)	90.5 (81.0,97.6)	92.9 (87.1,98.6)
GFR4	65.5 (51.8,77.6)	88.1 (78.6,97.6)	78.6 (68.6,88.6)

^a^GFR*ix* or GFR*i*: *i* and *x* implies the sampling time (hr) of the blood used to calculate the corresponding plasma clearance of iohexol. Units are in *ml/min/1.73m*^*2*^

^b^Accuracy was calculated as the percentage of estimate within 30% of the measured GFR (P_30_) and the percentage of estimate within 10% of the measured GFR (P_10_)

^c^As for accuracy, the value 100 represents none of the estimates differed from the mGFR by more than 30% or 10% with a 95% CI of (100,100)

## Discussion

In this paper, we have presented a comparison of 2 commonly used plasma sampling methods for GFR measurements: single- and dual-plasma sampling method. The data demonstrated well that DPSM using blood samples drawn at 2- and 4-h post-injection obtained the best correlation in terms of both precision and accuracy.

These results were highly consistent with the early study done by Waller DG [15], who compared alternative methods including SPSM, DPSM and external detector clearance rate using 2–5 h samples. The 2- and 4-h blood sampling correlates excellently with multiple-point plasma clearance (*r* = 0.996) with a standard error of 2.8 ml/min/1.73m^2^. Similar results were found by Russell et al. [22], who compared the DPSM technique with a two-compartment GFR assessment.

In fact, the BNMS guidelines [23] recommend a slope-intercept method requiring between two and four samples in the conclusion that “the majority of literature suggests that the single-sample method is less precise than the slope-intercept technique.” On the other hand, the plasma clearance measurement recommended by the current international guidelines [24] is the single-sample technique for clinical measurement of GFR in patients with GFR ≥ 30 ml/min/1.73m^2^ based on the Groth 4-h methodology [10]. However, both of the guidelines were written decades ago.

It should be noted from Table 1 that all the studied methods had a low bias and yielded optimal agreement with the 3 pt. iGFR, while the performance in precision and accuracy varied substantially regarding a certain method or GFR level (Table 2). This is partially because positive errors and negative errors cancel each other out, and thus blunt the real deviation. This phenomenon can be even more obviously observed at a lower GFR level and SPSM using a too-early sample (2-h SPSM, see Fig. 2). The explanation for this is well documented in the literature [4, 25, 26]: a terminal monoexponential clearance is not reached even at 4 h and therefore some AUC is missing from the calculation, causing the GFR to be overestimated [27]. However, DPSM suffers from the same deficiency rooted in the mono-compartment model, which results in a tendency towards overestimation when GFR was low and the opposite when GFR was high [28].

Prolongation of the sampling time is the main strategy for avoiding this error, which is crucial in SPSM. Indeed, in our study, by using a later blood sampling (4 h), the precision of SPSM was greatly improved as the IQR of the difference decreased from 21.99 to 4.93 ml/min/1.73m^2^, while the accuracy within P_30_ increased from 55.2 to 82.8%. Conventional practice to choose proper sampling time is based on the expected GFR before testing. Jacobsson calculated the optimal sampling time for clearance values around 100 ml/min to be 3 h and for clearance values around 30 ml/min to be 10 h [8]. For severe renal dysfunction (expected GFR < 15 ml/min/1.73m^2^), a blood sample drawing at 24 h is suggested [24]. Recently, a systematic review [27] compared SPSM results with a gold standard nine-point AUC measurement of GFR as well as slope-intercept methods (including the 2-, 3- and 4-h three-point plasma clearance and the 2- and 3-h DPSM) for 412 published GFR studies and concluded that the method described by Fleming [14] is the best SPSM and provides equivalent accuracy and precision to the slope-intercept-GFR.

The limitation of this study is that we lack data beyond 4 h, which will result in slightly overestimation of the GFR, as discussed above. Schwartz et al. [4] examined the plasma disappearance curve of iohexol in 27 children to determine the degree of overestimation in GFR due to shortening sampling time from 6 to 5 and 4 h. According to the multi-point AUC measurement, the authors found a significant 3% overestimation if sampling time was truncated at 4 h post-injection. However, the differences did not become much larger when only lower GFR values were examined and a 3% overestimation is probably clinically irrelevant as discussed by the authors. Moreover, it was demonstrated in their work that the area under the slow curve of the 4 h study was not significantly different from 6 h, which is to say, the AUC used to calculate DPSM-GFR was not significantly different between 4- and 6-h.

## Conclusions

In conclusion, clearance values based upon three-point plasma samples can be substituted by 2- and 4-h DPSM. When GFR was ≥60 ml/min/1.73m^2^, 4-h SPSM demonstrated approximate accuracy and thus can be recommended for clinical measurement of GFR. For patients with expected GFR < 60 ml/min/1.73m^2^, a prolonged sampling time is more reliable.

## Abbreviations

3 pt iGFR: The 2-, 3- and 4-h 3-point plasma clearance of iohexol
AUC: Area under the curve
BNMS: British Nuclear Medicine Society
BSA: Body-surface area
CCC: Concordance correlation coefficient
CI: Confidence interval
CKD: Chronic kidney disease
DPSM: Dual-plasma sampling methods
GFR: Glomerular filtration rate
HPLC: High-performance liquid chromatography
IQR: Interquartile range
mGFR: Measured glomerular filtration rate
SPSM: Single-plasma sampling methods
